# Acute Pneumonia Like Illness and Sepsis in India: Is it Time to Suspect Pulmonary Melioidosis?

**DOI:** 10.7759/cureus.36122

**Published:** 2023-03-14

**Authors:** Ananyan Sampath, M Sukumar, Penuboina Tejaswini, Ayush Gupta, Sagar Khadanga

**Affiliations:** 1 General Medicine, All India Institute of Medical Sciences, Bhopal, Bhopal, IND; 2 Microbiology, All India Institute of Medical Sciences, Bhopal, Bhopal, IND

**Keywords:** artificial intelligence, fever, acute pneumonia, melioidosis, burkholderia, chatgpt

## Abstract

This article describes a case of melioidosis, a severe and potentially fatal disease caused by the Gram-negative bacillus *Burkholderia pseudomallei*, in a 55-year-old female in India. The disease is endemic in Southeast Asia and Northern Australia. Recently there has been an increased number of cases reported in India. The source of *B. pseudomallei* in India is thought to be soil and water, with the most common mode of infection being through skin contact. The clinical presentation of melioidosis in India varies greatly, making diagnosis difficult. The case presented here with a history of acute febrile illness and progressive dyspnoea, with clinical worsening leading to intensive care unit (ICU) care. We managed this acute pneumonia-like melioidosis with antibiotics and supportive care which showed rapid recovery at follow-up. This case highlights the need for a high index of suspicion and increased awareness of early diagnosis of melioidosis in the Indian subcontinent to improve the patient.

## Introduction

*Burkholderia pseudomallei*, a previously rare Gram-negative bacteria, presents with a wide variety of clinical conditions congregated as melioidosis, a severe and potentially fatal disease caused [1]. The disease is earlier endemic majorly in Southeast Asia [2-3], and Northern Australia [4], now has a rising trend of detection in India and has also been reported in India in a few situations [5-7]. 

The source of *B. pseudomallei* in India is thought to be soil and water [2, 7]. The organism can survive in a variety of environmental conditions and can infect humans through multiple routes, including inhalation, skin contact, and ingestion. The most common mode of infection in India is through skin contact, often as a result of occupational exposure to contaminated soil or water. Still, other uncommon sources include rats, rabbits, Guinea pigs through routes of inoculation, inhalation, aspiration, or ingestion. These generally occur in patients with coexisting underlying conditions like diabetes mellitus, renal failure, and trauma or in farmers/animal breeders [8].

The clinical presentation of melioidosis in India can vary greatly, holding true to its eponym ‘great mimicker,’ with symptoms ranging from asymptomatic infection to severe sepsis and septic shock [1]. The most common symptoms include fever, cough, chest pain, and difficulty breathing. Other symptoms may include skin ulcers/abscesses, joint pain, septicemia, pneumonia, and neurological symptoms [4, 9-10]. Atypical presentations of melioidosis in India include chronic infections, which can present as chronic skin or soft tissue infections, or asymptomatic carriage. Rare forms of melioidosis in India include disseminated melioidosis, which can involve multiple organ systems, and neurological melioidosis, which can present with symptoms such as seizures and meningitis. These rare forms of the disease can be difficult to diagnose and treat and have a poor prognosis.

Herein, we present one such rare presentation of a 55-year-old female who presented to the emergency department (ED) in Madhya Pradesh (Central India) with a history of a week-long acute febrile illness associated with progressively worsening shortness of breath with no history of immunodeficiency or travel which presented a diagnostic challenge and had a near-fatal encounter.

## Case presentation

A 55-year-old female presented to the ED with history of an acute onset, intermittent high grade febrile illness for a week associated with gradually worsening shortness of breath and dry cough.

Upon general examination, the patient was conscious and oriented. Pulse rate was 120 beats per minute, systolic pressure of 134 mmHg, and respiratory rate was 22 breaths per minute, febrile at 102.3°F and oxygen saturation at 96% on room air. Arterial blood gas (ABG) showed compensated respiratory alkalosis. Complete blood count, renal and liver function tests, X-ray chest, and blood cultures were ordered and the patient was admitted to the High Dependency Unit. She was provisionally diagnosed to have a tropical fever and conservatively managed with Inj. ceftriaxone 1 g twice daily and oral doxycycline 100 mg twice daily.

Day 1: Hematological and serum laboratory investigations revealed neutrophilic leucocytosis (23,000 cells per mm3/82% predominance) with euvolemic hyponatremia (130 mmol/L). Within 24 h of admission, the patient deteriorated and was started on intravenous (IV) inotropic support for maintenance of mean arterial pressure at 65 mmHg. Inj. ceftriaxone dose was doubled and oral doxycycline was switched over to IV route of administration. Chest X-ray revealed bilateral lower lobe heterogenous opacification, presumed to be pneumonia.

Day 2: The patient deteriorated further, now requiring non-invasive ventilatory (NIV) support with sustained of low dose IV inotropes. Ceftriaxone was switched over with IV piperacillin-tazobactam 4.5 g every 6 h. Blood culture revered Gram negative bacilli with suspiciousness of pseudomonas-like organism.

**Figure 1:**
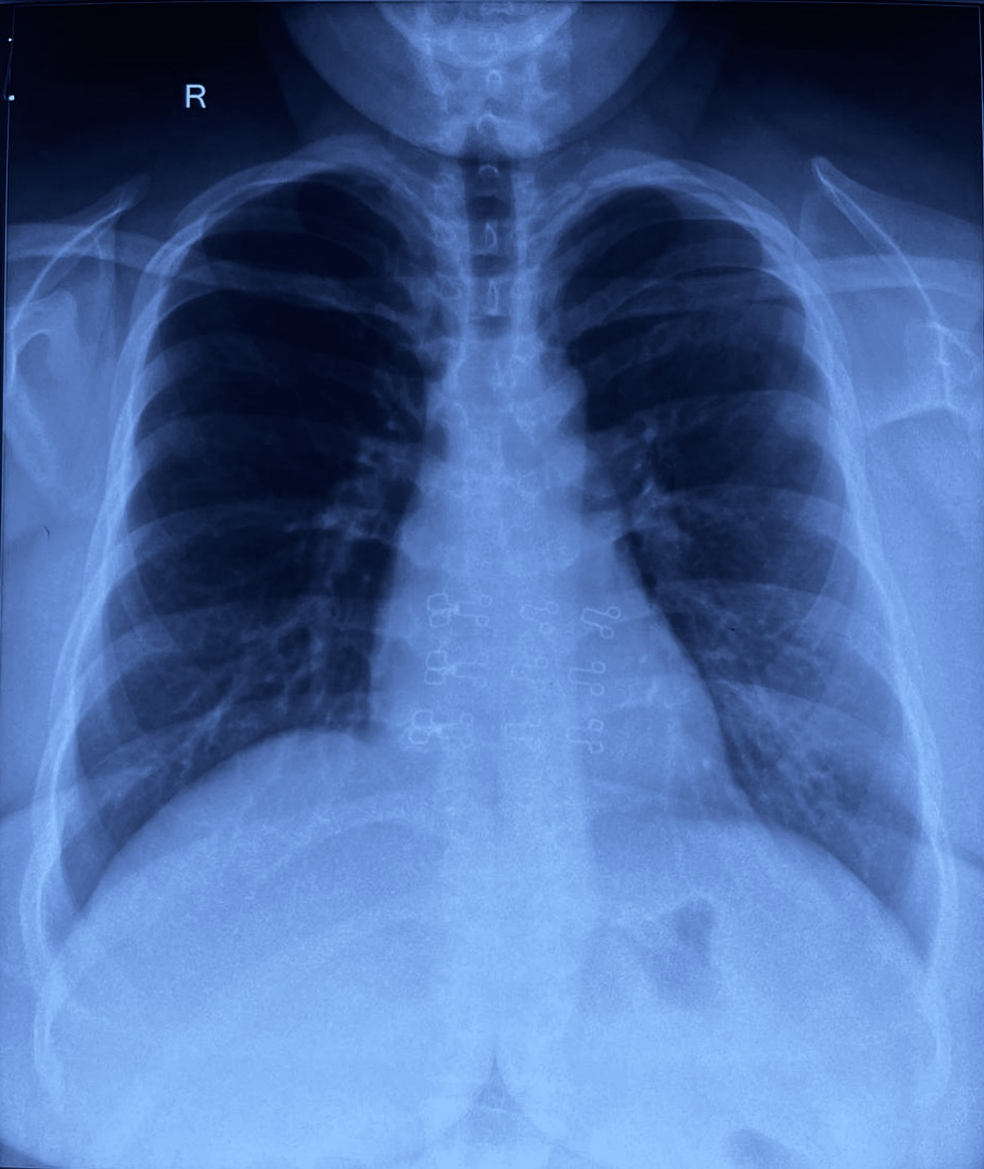
Chest X-ray indicative of bilateral heterogeneous opacities, indicative of pneumonia.

Day 3: The patient was difficult as she deteriorated further; NIV and positive pressure controlled mechanical ventilation were initiated to maintain breathing. The patient continued to have fever ranging up to 104°F and a repeat chest X-ray revealed worsening of congestion as shown in Figure 1. The IV antibiotic therapy was not altered.

**Figure 2:**
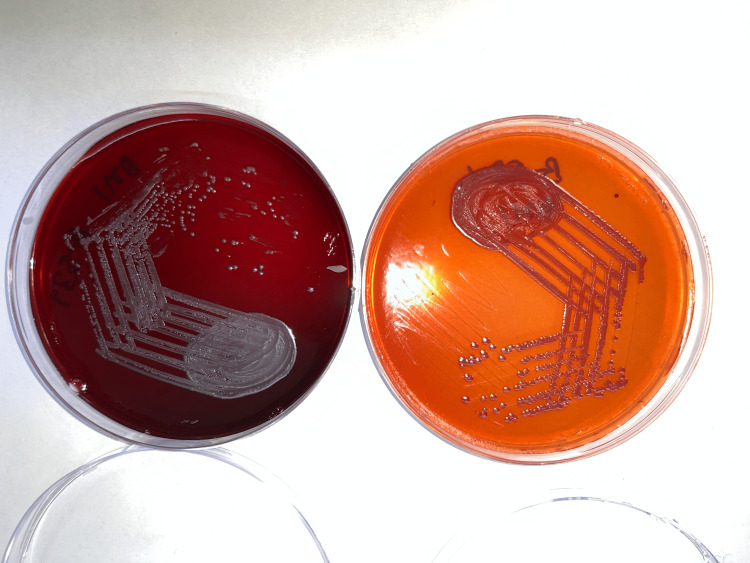
Culture plates of Burkholderia pseudomallei isolated from blood samples of patient in blood agar (left petri dish) and MaConkey agar (right).

Day 7: Burkholderia pseudomallei was identified from both the blood culture bottles which was sensitive to cotrimoxazole, ceftazidime, and meropenem. Piperacillin was switched over to Inj. meropenem 1 g three times daily, maintained on positive pressure mechanical ventilation NIV and IV vasopressors. Culture plates of samples retrieved from patient’s blood showing growth on blood and MacConkey agar as shown in Figure 2.

Day 10: The ventilatory support improved gradually and the patient was weaned of ventilatory support by day 12. The patient was maintaining SPO2 at 94% at 2 L/min of oxygen through nasal prongs and shifted to general ward from the ICU. The patient’s blood pressure was maintained without the necessity of vasopressors.

Day 15: The patient was ambulatory with no requirement of oxygen. The patient was lethargic with poor oral intake and was continued with intra venous meropenem 1 g three times daily with high calorie and high protein diet.

Day 20: General condition improved significantly and no complaints of fever but with occasional dry cough. Meropenem dose was decreased to 1500 mg a day in three divided doses along with oral cotrimoxazole (1600 mg/320 mg) was initiated in two divided doses.

Day 25: Meropenem was stopped and oral cotrimoxazole (1600 mg/320 mg) per day was continued. Folic acid 5 mg a day was also added to reduce the toxicities of cotrimoxazole. The patient was discharged from the hospital with advice to follow up as an outpatient after a week.

**Figure 3:**
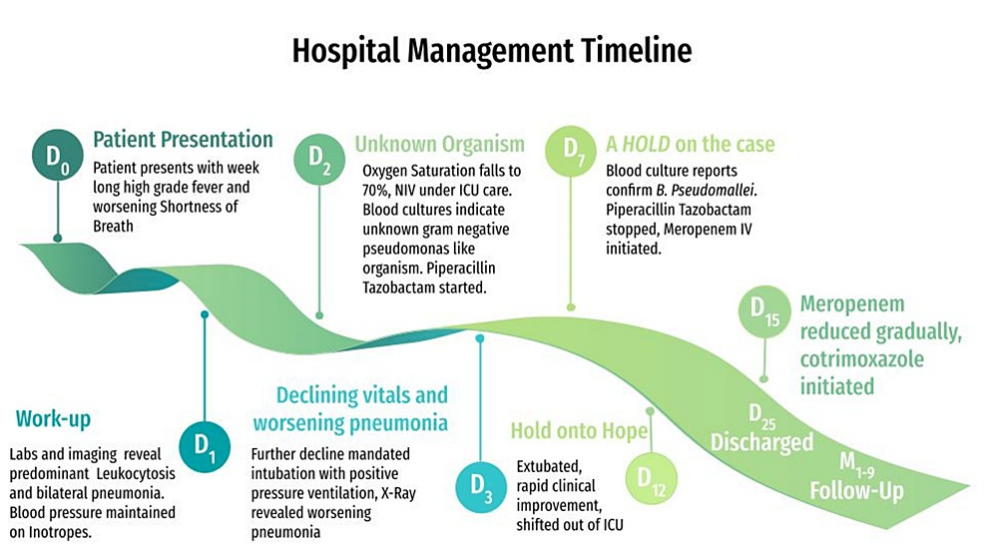
Case timeline from presentation to follow-up.

Follow-up: The patient was followed up weekly for a month and then monthly for 6 months. At 6 months of cotrimoxazole therapy, it was stopped. The patient was last seen at 9th month since presentation and was doing fine. A schematic representation of the case timeline is shown in Figure 3 for easier comprehension.

## Discussion

*Burkholderia pseudomallei* is an organism of growing concern in the recent few years in India, where an increased number of cases have been reported in the last decade, including our institute [2, 6-7, 11]. A saprophytic bacteria hosts a wide array of virulence factors such as the polysaccharide capsule, lipopolysaccharides, toxins, enzymes such as hemolysin, lipases and proteases, type III secretory systems, quorum sensing, type IV pilli and siderophores for iron stealing [12]. This arsenal of virulence factors enabled B. pseudomallei to be used as an agent of bioterrorism [13] and receive the eponym “Vietnam time-bomb disease” [14].

Due to the wide array of virulence factors, modes of infection and patient susceptibility, *B. pseudomallei* presents with a very vast variety of clinical presentations. A literature review by ChatGPT revealed three atypical cases of melioidosis across the decade. The presentations included chronic osteomyelitis [15], relapsing pneumonia [16] and melioidosis masquerading as a brain abscess with meningitis [9]. A thorough literature from various other medical databases revealed other presentations of melioidosis across the world with large case series conducted in Australia and Western India, most cases occurred in immunocompromised conditions or with underlying comorbid conditions or risk factors such as diabetes mellitus, renal failure, farmers or recent travel history to highly endemic areas as shown by literature but this case was unique to not have any of the above [8] and occurred in an immunocompetent host, similar to *Karuna et al.* [7].

Even though pneumonia is not an uncommon presentation, this case depicted no other common localizing symptoms such as cutaneous manifestations (ulcers, rash), or visceral abscesses and to the best of our knowledge, is one of the first few cases of acute pneumonia in the Southeast Asian region. Acute pneumonia with sepsis requiring invasive mechanical ventilation is an unusually rare presentation in a previously immunocompetent patient. This case is being documented to normalize routine blood cultures in cases of high index suspicion in similar acute respiratory tract-like infections even in immunocompromised individuals further in India.

## Conclusions

Melioidosis is a severe and potentially fatal disease caused by *B. pseudomallei* that has been reported in the Indian subcontinent. The clinical presentation of melioidosis in this region can vary greatly, with atypical presentations making diagnosis difficult. The case is presented here because high index of suspicion is required for melioidosis, even in young patients with no travel history or without any known co-morbidities. Early diagnosis is of paramount importance in managing the case with improved outcomes. 

## Figures and Tables

**Figure 4:**
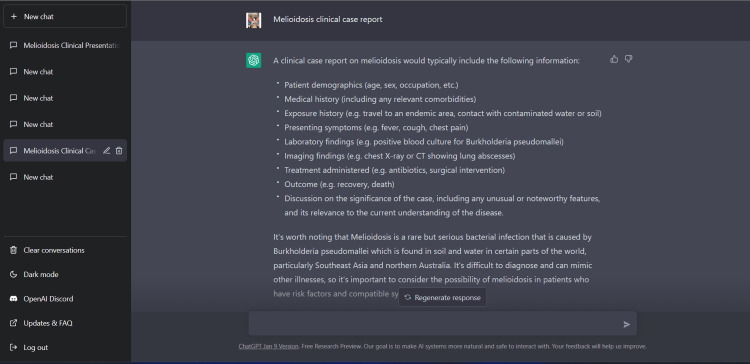
Depicting the content necessary for population of a case report on melioidosis.

**Figure 5:**
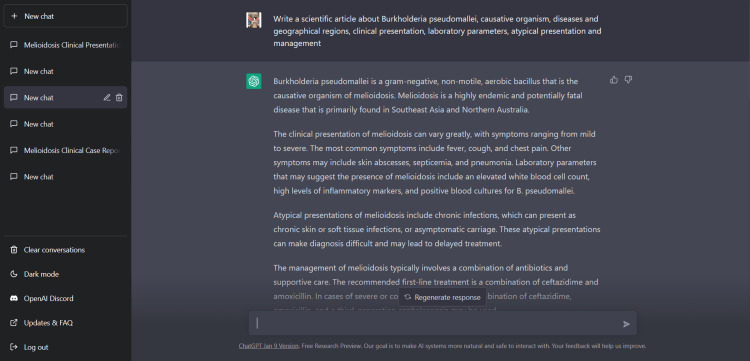
Representation of the introduction search performed using ChatGPT.

## Appendices

ChatGPT was used to ideate the case report writing headings and further to elucidate the introduction and perform a brief literature review for the article as shown below in supplementary figures below (Figures 4-5).
